# Changes in Hematological and Biochemical Profiles in Ovariohysterectomized Bitches Using an Alfaxalone–Midazolam–Morphine–Sevoflurane Protocol

**DOI:** 10.3390/ani12070914

**Published:** 2022-04-02

**Authors:** Mónica Rubio, Katiuska Satué, José María Carrillo, Ángel Hernández Guerra, Belén Cuervo, Deborah Chicharro, Elena Damiá, Ayla Del Romero, Joaquín Sopena

**Affiliations:** Department of Animal Medicine and Surgery, Faculty of Veterinary Medicine, CEU-Cardenal Herrera University, 46115 Valencia, Spain; mrubio@uchceu.es (M.R.); jcarrill@uchceu.es (J.M.C.); angelhdez@uchceu.es (Á.H.G.); belen.cuervo@uchceu.es (B.C.); debora.chicharro@uchceu.es (D.C.); elena.damia@uchceu.es (E.D.); ayla.delromero@uchceu.es (A.D.R.); jsopena@uchceu.es (J.S.)

**Keywords:** alfaxalone, biochemistry, dog, hematology, ovariohysterectomy

## Abstract

**Simple Summary:**

Laboratory tests are an integral part of the overall diagnostic procedures for both physiological and pathological conditions. Ovariohysterectomy (OHE) in bitches is an invasive procedure, producing moderate to severe pain. The response to surgical stress is characterized by a series of neuroendocrine and metabolic changes that can be represented on hematological and biochemical profiles. The objective of this study was to evaluate the physiological response to surgical stress, predict the presence of possible post-surgical complications, and where appropriate, establish the appropriate treatment based on the hematochemical changes during and after OHE in healthy bitches. OHE in this study induced transient changes in certain hematological and biochemical parameters. Knowledge of the hematochemical changes in response to stress and trauma induced by OHE in healthy dogs would allow to predict possible post-surgical complications.

**Abstract:**

The aim of this study was to monitor hematochemical changes during and after OHE in bitches. Twenty-four females were anesthetized with alfaxalone, midazolam, morphine and sevoflurane. Blood samples were taken before anesthesia (T0), at 30 (T1), and 60 min (T2), at 3 (T3), 6 (T4), 12 (T5), and 24 h (T6), and at 3 (T7) and 7 days (T8) from the start of surgery. Red blood cells (RBC) and packed cell volume (PCV) decreased significantly from T1 to T5 and hemoglobin (HB) concentration from T4 to T6. Both the white blood cell (WBC) and neutrophil (NFS) count increased significantly from T3 to T6, monocyte (MON) from T2 to T5, and eosinophil (EOS) at T5. Platelet (PLT) and plateletcrit (PCT) significantly decreased at T5 and increased from T6 to T8; platelet distribution width (PDW) increased significantly from T3 to T6. Creatine kinase (CK) activity increased significantly from T5 to T7. Glucose (GLU) concentrations increased significantly at T2 and P from T2 to T3. TG levels decreased from T2 to T4 and blood urea nitrogen (BUN) levels from T1 to T7, subsequently increasing until T8. Changes possibly resulting from stress and surgical trauma, as well as hemodilution and splenic storage, are due to anesthesia and surgery. In healthy bitches, these changes tend to gradually stabilize after the ending of OHE. A post-operative follow-up is essential to detect possible post-operative complications.

## 1. Introduction

Ovariohysterectomy (OHE) is one of the most common surgical contraceptive techniques used for population control in bitches. Other potential benefits of sterilization include prevention of inherited diseases, elimination of undesirable behaviors associated with hormonal cycling and if performed before 2½ years of age, a reduction in the risk of ovarian, uterine, and mammary neoplasia [1]. Other pathologies that justify OHE include uterine and ovarian tumors, pyometra and glandular cystic uterine hyperplasia with secondary infection leading to chronic metritis [2,3]. For these clinical purposes, various surgical techniques, including traditional midline, lateral flank and laparoscopic OHE are used [4].

Surgical stress response during and after surgery involves a physiological reaction of the organism to restore homeostasis or counteract the stimulus [5]. Somatic and autonomic afferent nerve impulses generated at the injury site activate the endocrine response mediated by the stimulation of the sympathoadrenal system and the hypothalamic-pituitary-adrenal axis [6]. At the same time, inflammatory and immune responses begin to develop, mediated by cytokine secretion products of activated leukocytes, fibroblasts and endothelial cells. These compensatory mechanisms prevent secondary damage and increase the availability of substrates required by essential organs and healing tissues [7,8] and implicate hematologic, metabolic, and immunomodulatory changes [8]. Several studies have evaluated the changes of hematochemical parameters in OHE bitches following various surgical techniques, and using various anesthetic protocols, providing different results [9,10,11,12].

Surgical anesthesia is characterized by immobility, absence of consciousness, muscle relaxation and lack of pain [13]. It has been suggested that the application of any anesthetic can potentially modify the physiological response to the surgical procedure due to cardiovascular, respiratory, digestive, and neuroendocrine alterations, thus, causing homeostatic, metabolic, and immunological changes [8,14]. Nevertheless, there is controversy about this since a single anesthetic administration by itself has shown to cause scarce or no response to stress [15]. Therefore, most metabolic changes appear to be caused by the surgical procedure itself [16].

Alfaxalone (3-alpha-hydroxy-5-alpha-pregnane-11, 20-dione) is a synthetic neuroactive steroid molecule that modulates the gamma-aminobutyric acid A (GABA_A_) receptor and causes neuro-depression, muscular relaxation, chemical restraint and/or general anesthesia [17]. It can be used for sedation, induction of anesthesia, and for total intravenous anesthesia (TIVA). It causes a minimal change in cardiac output or blood pressure when clinically relevant doses are administered to healthy patients [17]; it has a high therapeutic index, is short-acting, and is noncumulative [18,19]. These characteristics make alfaxalone ideal for its use as an induction agent or injectable anesthetic. Alfaxalone is used for OHE in bitches [20,21,22,23]. Midazolam, administered before anesthetic induction, provides good muscle relaxation, reducing the alfaxalone-related hyperkinesia [24,25]. Sevoflurane, due to its low blood:gas partition coefficient and rapid onset of action, allows easy control of the anesthetic depth [26]. Finally, for its efficacy in treating intraoperative and post-operative pain, morphine continues to be used in veterinary medicine [26], even though other drugs such as methadone and fentanyl are also being widely used in many countries.

At present, the physiological importance of blood findings in bitches undergoing OHE in both clinical and experimental studies is not well defined. Although the incidence of complications in OHE procedures is relatively low in healthy animals, it still involves risks during and after surgery [27]. Accordingly, the aim of the present study was to monitor hematologic and biochemical changes during and after OHE in healthy bitches following the administration of alfaxalone, midazolam, and morphine as premedications, alfaxalone as induction, and sevoflurane as maintenance. The exploration of dynamic changes in normal hematochemical tests could help to better understand possible complications during and after OHE and to establish the most effective treatment as soon as possible.

## 2. Materials and Methods

### 2.1. Animals

This study was conducted in the Veterinary Teaching Hospital of CEU-Cardenal Herrera University (Valencia, Spain). A total of 24 client-owned adults, healthy, mixed-breed female dogs were included. A complete history of the animals, including breed, age, parity and stage of the oestrous cycle, was recorded. After being informed of the fundamentals of the study, the owners signed a consent form for their pets to be included. All animals underwent a pre-anesthetic evaluation to confirm that they were healthy. This evaluation included physical examination, complete hematological and biochemical analysis, thoracic X-rays, and electrocardiogram. In addition, all animals were subjected to a commercially available SNAP^®^ 4Dx^®^ Plus test for the detection of antigen to Dirofilaria immitis, antibodies to Borrelia burgdorferi, Anaplasma phagocytophilum, Anaplasma platys, Ehrlichia canis and Ehrlichia ewingii (IDEXX SNAP^®^), yielding negative results in all cases. Only animals classified as ASA I were included in the study. None of the animals received medical treatment during the last 6 months prior to the study, nor were they diagnosed with active disease. During the study period, any animal received medication, and no animals had to be excluded.

### 2.2. Anesthetic and Surgical Procedure

Animals were received and placed in individual accommodation 24 h before surgery with water and food ad libitum. The fasting time was 8 h of solids and 2 h of liquids. No animals received any medications prior to surgery. All the anesthetic procedures were performed by the same veterinary anesthesiologist. After placement of a cephalic 18 gauge 1” catheter, the anesthetic protocol consisted of premedication with 5 mg/kg IM alfaxalone (Alfaxan^®^ 10 mg/mL; Dechra Veterinary Products SLU, Barcelona, Spain), applied at two different points to avoid patient discomfort, in combination with 0.1 mg/kg IM midazolam (Midazolam Hospira^®^ 5 mg/mL, Roche, Madrid, Spain) and 0.2 mg/kg IM morphine (Morphine Chloride 2%^®^, B-Braun, Melsungen, Germany). Induction consisted of alfaxalone administered through the cephalic catheter and titrated to effect until tracheal intubation was possible with an appropriately sized cuffed endotracheal tube. Maintenance was conducted with sevoflurane (Sevoflo^®^, Esteve Veterinary, Barcelona, Spain) vaporized in a mix of 60% oxygen and 40% air, as needed. During the surgical procedure, all animals received IV fluid therapy with Ringer-Lactate solution (5 mL/kg/h, Braun^®^, Barcelona, Spain). Intraoperative monitoring consisted of body temperature, electrocardiogram, capnography, pulse oximetry, noninvasive blood pressure and oxygen and vapor concentrations. Intermittent positive pressure ventilation (VPPI) was applied when necessary to ensure normocapnia, and the volume was regulated to keep the end-tidal CO_2_ at normal levels (35–45 mmHg) [28]. Post-operative care consisted of hospitalization during the first 24 h and analgesia receiving morphine (0.2 mg/kg/6 h for 24 h).

All surgeries were performed by the same surgeon with the help of an assistant. Thirty minutes before starting the surgery, a single dose of cefazolin was administered to all animals (20 mg/kg/IV, Cefazolina Normon^®^, Normon laboratories, Madrid, Spain) [29]. The surgical area was clipped and scrubbed. The distance from xiphoid to pubis was measured to calculate the size of the incision (20% of this length) and to standardize the surgical wound. Surgery was performed by a horizontal right flank approach, according to a published procedure [30]. A single dorsoventral incision in the skin was made on the right flank of the animal, starting caudal to the midpoint between the last rib and iliac crest. The subcutaneous tissue was dissected and the fibers from the muscle bellies of the external abdominal oblique, internal abdominal oblique and transverse abdominal were opened separately, according to their fiber direction, as described in the literature. The suspensory ligament was sectioned, the ovarian artery and vein and the uterine artery and vein were double ligated, and the uterus excised. For closure, muscles were sutured in two layers with the transverse and internal oblique muscle together, and the external oblique muscle as a second layer, as described in the literature [30]. Subcutaneous tissue was sutured with a simple continuous pattern, and the skin was closed with an intradermal pattern. For all layers, an absorbable monofilament glyconate suture was used (Monosyn^®^, B. Braun VetCare SA, Barcelona, Spain). No blood loss was observed in any of the animals and no post-operative complications occurred.

### 2.3. Blood Sampling

Blood samples were obtained from patients at different study times: before anesthesia (T0), at 30 min (T1), 60 min (T2), 3 h (T3), 6 h (T4), 12 h (T5), 24 h (T6), 3 days (T7) and 7 days (T8) after the anesthetic and surgical procedure. A total volume of 1.5 mL of blood was extracted from each animal, of which 0.5 mL was anticoagulated in EDTA (Tapval^®^, Barcelona, Spain) and 1.0 mL in lithium heparin (Tapval^®^, Barcelona, Spain) for hematological and biochemical analysis, respectively. The anticoagulated samples in lithium heparin were centrifuged at 3000 rpm for 10 min (Centronic model centrifuge, Barcelona, Spain) to obtain plasma. All samples were analyzed immediately after extraction.

### 2.4. Analytical Methods

Both hematological and biochemical analyses were performed at different study times. The hematological parameters included were: red blood cell count (RBC × 10^12^/L), hemoglobin concentration (HB; g/dL), hematocrit (PCV; %), medium corpuscular volume (MCV; fL), mean corpuscular hemoglobin (MCH; pg), mean corpuscular hemoglobin concentration (CMCH; g/dL), erythrocyte distribution width (RDW; %), reticulocytes (RET;/µL); white blood cells (WBC; ×10^3^/L), neutrophils (NFS; ×10^3^/L), lymphocytes (LYMPH; ×10^3^/L), eosinophils (EOS; ×10^3^/L), monocytes (MON; ×10^3^/L), and platelet counts (PLT; ×10^3^/L), plateletcrit (PCT; %), mean platelet volume (MPV; fL) and platelet distribution width (PDW; %). The parameters studied in the serum biochemistry were: total plasma proteins (TPP; g/dL), albumin (ALB; g/dL), globulins (GLOB; g/dL) and glucose concentration (GLU; mg/dL); alanine aminotransferase (ALT; UI/L), alkaline phosphatase (ALP; UI/L), aspartate aminotransferase (AST; UI/L), gamma glutamyl transferase (GGT; UI/L) and creatine kinase (CK; UI/L) activities; total bilirubin (TBIL; mg/dL), triglycerides (TG; mg/dL), cholesterol (CHOL; mg/dL), calcium (Ca; mg/dL), phosphorus (P; mg/dL) and sodium (Na; mEq/L), chloride (Cl; mEq/L), potassium (K; mEq/L) as electrolytes concentrations. Hematological determinations were carried out with the cell counter CELL-DYN Emerald (Abbot, Hanover, Germany) and for the analysis of biochemical parameters, the automatic system Spin 200E was used (Spinreact SAU, Barcelona, Spain).

### 2.5. Statistical Analyses

Statistical analyses were conducted using SPSS (IBM SPSS Statistics for Windows, Version 23.0, IBM Corp, Armonk, NY, USA) statistical software. To analyze the existence of significant differences between the times considered (T0, T1, T2, T3, T4, T5, T6, T7 and T8), the Kruskal–Wallis test was used. By means of the Mann–Whitney test, significant differences among times were assessed. Results are given as mean ± SD. A *p*-value < 0.05 is considered significant.

## 3. Results

The mean age, mean weight and mean body condition of the animals included in the study were 5 ± 2 years, 20.3 ± 7.2 kg, and 4–5 (according to the 9-point scale), respectively [31]. The required dose of alfaxalone was 3.1 ± 0.9 mg/kg. Compared to baseline levels, the RBC count and PCV decreased significantly from T1 to T6 (*p* < 0.05) and the HB concentration decreased from T4 to T6 (*p* < 0.05; Table 1). The WBC and NFS count increased significantly from T3 to T6 (*p* < 0.05), and the MON count increased significantly from T2 to T5 (*p* < 0.05). In addition, a significant increase in EOS count was seen at T5 (*p* < 0.05; Table 1). Compared to T0, the PLT count and PCT significantly decreased at T5, followed by a significant increase from T6 to T8 (*p* < 0.05), and the PDW significantly increased from T3 to T6 (*p* < 0.05; Table 1). No significant differences were observed in MCV, MCH, MCHC, RDW, RET, LYMPH and MPV, ranging between 61.1–73.8 fL, 18.2–22.3 pg, 29.0–33.0 g/dL, 11–15%, 0.4–8.9/µL, 1.10–7.40 ×10^3^/L and 3.80–9.10 fL, respectively.

Regarding biochemical parameters, GLU concentration increased significantly at T2, remaining elevated until T4. A progressive and significant decrease in BUN levels from T1 to T7 was observed, followed by a progressive increase until the end of the study, the time when BUN reached similar values to the baseline. The TG concentration decreased significantly from T2 to T4 (*p* < 0.05; Table 2), without subsequent changes. Plasma CK activity increased significantly after T5, remaining elevated until T7 (*p* < 0.05; Table 2). Compared to baseline values, *p* concentration increased significantly from T2 to T3 (*p* < 0.05; Table 2). No differences were found in TPP, ALB, GLOB and the ALB/GLOB ratio, CHOL, CREAT, ALP, GPT, TBIL, Ca and electrolytes (Na, K and Cl). TPP, ALB, GLOB concentrations and the ALB/GLB ratio ranged between 5.24–8.48 g/dL, 2.63–4.90 g/dL, 2.15–6.43 g/dL and 0.28–2.12, respectively. CHOL and CREAT concentrations, ALT and ALP enzyme activities and TBIL concentrations fluctuated from 79–291 mg/dL, 0.49–1.61 mg/dL, 20–82 IU/L and 38–202 IU/L and 0.1–0.9 mg/dL, respectively. The concentrations of Na, K, Cl and Ca varied between 139–157 mmol/L, 3.0–5.6 mmol/L, 106.0–120.0 mmol/L and 7.20–11.6 mg/dL, respectively.

## 4. Discussion

In this study, RBC and PCV levels decreased from 30 min to 24 h, also the HB concentration decreased from 6 to 12 h after surgery, followed by an increase to baseline levels. Similar results in relation to RBC and PCV were obtained in other studies [9,10,11,32] during intra and post-surgical times when different surgical and anesthetic protocols were used. The similarity between results suggests that erythrocyte parameters respond in a similar way despite the anesthetic and surgical protocols used in bitches that underwent OHE.

Decreased RBC and PCV can have different origins. First, it could be due to the accumulation of blood in the spleen after the administration of anesthetic agents [33]. Although the application protocol differs considerably from that used in this study, the administration of a single IV bolus of alfaxalone resulted in a significant increase in canine splenic volume from 0.17 L at baseline to 0.24 L after 15 min, and 0.23 L after 30 min. In the same study, there was a decrease of PCV from 46.3% at baseline to 40.6% after 15 min and 41.7% after 30 min [34]. Splenomegaly, due to alfaxalone administration in dogs, could be secondary to changes in smooth muscle tone and changes in systemic blood pressure and cardiac output, which alters the blood flow and blood loss [35] as well as to hemodilution in response to fluid therapy to preserve the blood flow in vital organs (brain, heart, liver and kidney) at the expense of other organs such as the skin and pancreas [10,36]. Second, the decline of PCV could also be the result of the sequestration of RBC in nonsplenic sites [37]. It is important to note that many events that commonly occur during surgery, such as blood loss, tissue hypoperfusion/ischemia, hypoxia and intraoperative fluid therapy, among others, can alter hematology, and should not be ignored. On the contrary, other studies showed no significant differences among erythrocyte parameters along OHE in bitches [12].

A temporary increase in WBC and NFS from 3 to 24 h, and in the MON count from 60 min to 12 h after the beginning of the OHE, without modifications in LYMPH counts, was observed in this study. These results partially confirm those obtained by other researchers using different anesthetic protocols and the same surgical technique. One study reported similar intraoperative increases in WBC and NFS, with LYMPH decrease [32]. The intraoperative and post-surgical neutrophilia and lymphopenia might have been the result of the stress caused by the anesthetic drugs, surgical trauma and the subsequent stimulation of the adrenal cortex [38]. It is well documented that OHE inflicts pain and stress as a result of tissue trauma, organ manipulation and inflammation [32]. Hancock et al. [39] reported a significantly higher peak of plasma cortisol levels in dogs 2 h after OHE. In response to the stress hormone, NFS shifts from the marginated to the circulating NFS pool. This neutrophilia might also be enhanced by the release of NFS from the bone marrow storage pool and the decreased migration of NFS to the tissues [40]. Although the transient mature neutrophilia in this study could be related to stress, no lymphopenia and eosinopenia were observed at any study time.

Contrary to our results, other studies revealed no significant decrease in WBC [12] or a slight decrease for a short time during anesthesia, after OHE in bitches [11]. It is known that the administration of α2-agonists suppresses the circulating catecholamines, which exerts a modulatory effect on leukocyte subpopulations [41]. In addition, dissociation agents also reduce leukocyte counts [42]. For example, ketamine and butorphanol produce an analgesic effect, reducing the response to stress by reducing cortisol and adrenaline and, consequently, the number of leukocytes. Reports of a decreased WBC post-operatively compared to the baseline, with negligible changes in NFS counts, are also available. These insignificant changes in NFS can be attributed to the effects of dexmedetomidine, which directly (by inhibiting the neuroendocrine response) or indirectly (through sedation and analgesia) obstruct the stress response when administered systemically. However, decreased WBC has also been related to hemodilution [10].

PLT count and PCT decreased at 12 h, and increased from 24 h to 7 days, whereas PDW increased from 3 to 24 h, without changes in MPV. The investigations regarding perioperative PLT counts in small animals are scarce and controversial. In some studies, there are higher PLT counts after OHE than pre-OHE in bitches [9]. PLTs are an acute-phase reactant that increases in response to systemic inflammatory processes because of increased pro-inflammatory cytokines, such as interleukins (IL-1, IL-6 and IL-11). Thus, the increase in PLTs and PDW observed in this study could be related to the systemic response and bleeding secondary to OHE. In addition, during the surgical inflammatory process, the release of activated platelets increases [43]. On the other hand, after sedation with acepromazine and atropine, a significant reduction in the PLT count and PLT aggregation capacity in response to ADP has been observed, without clinical signs of hemorrhage [44]. The decrease in the PLT count has been related to hemodilution during anesthetic procedures, due to vascular pooling as a consequence of vasodilatation or sequestration of blood cells in the spleen and lungs during the procedure [45]. Finally, one study reports no changes in PLT between pre- and post-operative counts in bitches undergoing OHE [46].

In this study, plasma GLU increased at 60 min, remained elevated until 3 days, and decreased thereafter. Similar results have been documented in dogs undergoing OHE [12,47]. The maximum GLU levels in the latter studies were observed 1 h after anesthetic premedication, and levels were back to baseline after 24 h post-anesthesia. In other studies, using a protocol consisting of medetomidine, carprofen, propofol and isoflurane for OHE, a significant increase in GLU levels was found at 1 and 6 h, and a decrease in GLU values at 24 h [48]. Interestingly, stabilization of GLU levels in the present study occurred later than in previous studies. However, in bitches anesthetized with dexmedetomidine and subjected to conventional OHE through the ventral midline, GLU levels increased significantly over the baseline values in the post-operative period [36].

It is documented that most anesthetic protocols induce a hyperglycemic state [49,50,51,52,53]. This hyperglycemia varies according to the mechanisms of action of the different anesthetic drugs. Thus, xylazine and α2-agonists (medetomidine and dexmedetomidine) cause insulin suppression and/or stimulation of glucagon release [54]; propofol induces decreased GLU transport and GLU utilization by tissues, impaired insulin, and increased adrenocortical hormones in dogs [55]. In addition, there is a failure of the usual cellular response to insulin (insulin resistance), which occurs in the perioperative period [49].

OHE is considered a moderate to severely painful and invasive procedure [56,57]. Surgical trauma leads to a stress response and increases muscular activity caused by damage to superficial nerve endings [58], stimulating the corticotropic releasing hormone (CRH). Following the stimulation of the hypothalamus, CRH stimulates adrenocorticotropin hormone (ACTH) and induces the adrenal gland to release cortisol. Furthermore, both the surgical stress and the anesthetic protocol lead to the altered endocrine secretion of insulin antagonists such as growth hormone [51] The latter, together with cortisol, trigger glycogenolysis in muscles and liver [59]. Mild-to-moderate stress hyperglycemia is protective because it provides a source of fuel for the immune system and the brain at a time of stress [60]. In addition, the activation of the sympathoadrenal system releases adrenaline, noradrenaline and glucagon. Delayed GLU metabolism and utilization, gluconeogenesis, lipolysis and insulin resistance culminate in an elevated plasma GLU concentration [8]. Intraoperative and post-operative hyperglycemia, in this study, could be due to the combined effect of the anesthetic and the surgical procedures. It is possible that alfaxalone may help induce a state of hyperglycemia. However, in other clinical procedures, no changes in blood GLU were detected when the same anesthetic was used [61].

CREAT values did not increase in the initial intervals after the administration of the agents, although BUN concentrations decreased from 30 min to 3 days [10]. Similar results for CREAT were observed for the same surgical procedure in the literature [32]. BUN levels progressively decreased from 30 min to 3 days, increasing progressively until the end of the study. The early decrease in BUN in these animals could be related to a lower protein intake prior to surgery or increased IV fluids. Taking into account that neither CREAT levels nor electrolytes (Na, K, Ca and Cl) concentrations change in the present study, any sign of renal dysfunction was ruled out. However, compared to the basal level, P concentration increased significantly from 60 min to 3 h, although none of the values were above the reference range. Hyperphosphatemia may result from reduced renal excretion secondary to decreased glomerular filtration rate (GFR) and is seen in cases of azotemia, which was not the case in this study [62].

TG concentration decreased from 60 min to 6 h, without modifications in CHOL. Ketamine-xylazine administration in dogs has been shown to induce a decrease in TG before and during anesthesia, related to falls in cholinesterase activity [63]. On the other hand, hepatic TG synthesis decreases by 50% in rats during oophorectomy [64]. Conversely, Bilen [65] and Chagas et al. [66] showed an increase in TG in dogs after OHE without modifications in CHOL. This elevation of TG is considered a physiological and transient condition that decreases at 7–12 h and could be due to food restriction prior to surgery.

CK activity increased almost 5-fold from pre-surgery values from 12 h to 3 days, subsequently decreasing to the baseline. CK is an enzyme found predominantly in skeletal muscle, and significantly elevated serum activity is largely associated with muscle damage. The marked increase in plasma CK in this study could be explained by the muscle damage produced during the flank approach. Earlier increases of this enzyme have been reported from 4 to 12 h [67] and, also at 48 h [68] post-OHE, and are probably proportional to the extent of muscle injury. The sustained increase in CK to 3 days in this study leads the authors to believe that CK is indeed a factor that can help measure stress and pain in these animals, despite the fact that OHE through the linea alba has been shown to cause minimal muscle trauma [67].

TPP, ALB, GLOB and the ratio ALB/GLOB did not change through the study time Similar results were obtained by Del Romero et al. [28] in dogs submitted to an ovariectomy (OVE) using a similar anesthetic protocol when studying ALB levels at 1, 24, 72 and 168 h post-intervention. However, hypoproteinemia and hypoalbuminemia have been reported to be the most notable changes in response to surgery. Indeed, the type and duration of surgical trauma [69,70] are related to increased protein catabolism in response to inflammation and/or infection. Both phenomena led to the production of immunoglobulins and acute-phase reaction proteins [71]. Furthermore, a decreased production of ALB by the liver and an increased vascular permeability may affect ALB levels [72]. The stability of TPP, ALB and GLOB could be explained by the good hydration status of the animals during surgery. In the same way, the absence of changes in the enzyme profiles of ALT, AST and TBIL determined, rules out the presence of possible hepatobiliary damage. The fact that the ALP levels did not change could be explained by the absence of bone or kidney damage due to the analgesic and anesthetic protocols used in this study.

A strength of this study was the fact that samples were obtained from healthy bitches with similar clinical histories, following the same anesthetic protocol and without post-operative complications, which allowed the authors to obtain representative and consistent results. However, a limitation of the study could be that some events that can occur during OHE and that are difficult to assess (such as the quality of tissue trauma, short stages of local hypoperfusion/ ischemia, or the release of some catecholamines, among others), could potentially affect some of the hematochemical results reported here.

## 5. Conclusions

In conclusion, conventional OHE in bitches using an alfaxalone-midazolam-morphine-sevoflurane protocol induces limited changes in certain hematological and biochemical parameters. Hematochemical analysis is a sensitive tool used to monitor the health status and surgical stress of the patient, as well as to detect any possible intra- or post-surgical complications.

## Figures and Tables

**Table 1:** Pre-, intra- and post-OHE mean ± SD of erythrocyte, leukocyte and platelets parameters in healthy bitches. Letter indicates significant differences vs. baseline values: ^a^ *p* < 0.05.

	RBC (×10^12^/L)	HB (g/dL)	PCV (%)	WBC (×10^3^/L)	NFS (×10^3^/L)	EOS (×10^3^/L)	MON (×10^3^/L)	PLT (×10^3^/L)	PDW (%)	PCT (%)
T0	6.87 ± 0.33	13.9 ± 0.98	44.7 ± 2.61	9.49 ± 1.74	4.86 ± 2.51	0.55 ± 0.30	0.48 ± 0.23	256.7 ± 69.34	16.2 ± 1.19	0.17 ± 0.05
(baseline)	(6.43–7.43)	(12.2–15.3)	(39.2–48.0)	(6.20–13.3)	(3.0–15.4)	(0.10–1.30)	(0.10–0.80)	(192–403)	(15–19)	(0.04–0.25)
T1	6.33 ± 0.44 ^a^	12.8 ± 1.38	38.8 ± 4.48 ^a^	9.80 ± 2.24	4.87 ± 1.82	0.39 ± 0.24	0.31 ± 0.24	236.7 ± 69.34	16.4 ± 1.12	0.15 ± 0.05
(30 min)	(6.00–7.50)	(11.1–16.7)	(32.0–49.6)	(6.40–14.1)	(3.36–10.4)	(0.10–0.90)	(0.0–0.80)	(192–403)	(15–21)	(0.04–0.26)
T2	6.05 ± 0.43 ^a^	12.8 ± 1.11	38.2 ± 2.87 ^a^	8.83 ± 1.95	4.31 ± 1.63	0.37 ± 0.24	0.28 ± 0.21 ^a^	249.1 ± 47.33	15.6 ± 1.00	0.15 ± 0.04
(60 min)	(5.46–6.90)	(11.8–16.0)	(31.9–43.6)	(5.70–12.0)	(2.53–8.48)	(0.0–1.10)	(0.0–0.80)	(190–325)	(14–18)	(0.04–0.24)
T3	5.96 ± 0.29 ^a^	13.0 ± 0.89	39.3 ± 2.97 ^a^	8.44 ± 2.33 ^a^	7.87 ± 3.32 ^a^	0.35 ± 0.37	0.59 ± 0.42 ^a^	251.5 ± 49.50	16.07 ± 0.97 ^a^	0.15 ± 0.05
(3 h)	(5.40–6.40)	(11.6–15.3)	(30.0–43.0)	(5.70–14.8)	(3.00–13.44)	(0.0–1.20)	(0.0–1.70)	(197–335)	(15–19)	(0.07–0.25)
T4	5.88 ± 0.33 ^a^	12.7 ± 0.98	38.1 ± 3.60 ^a^	12.6 ± 3.25 ^a^	10.09 ± 3.77 ^a^	0.45 ± 0.50	0.63 ± 0.30 ^a^	250.0 ± 52.4	16.7 ± 1.44 ^a^	0.14 ± 0.04
(6 h)	(5.10–6.32)	(11.0–14.8)	(30.0–42.9)	(5.80–17.4)	(3.40–16.8)	(0.10–2.20)	(0.10–1.20)	(190–350)	(14–20)	(0.04–0.21)
T5	5.85 ± 0.33 ^a^	12.4 ± 0.95	38.6 ± 3.65 ^a^	14.9 ± 4.83 ^a^	10.4 ± 4.10 ^a^	0.56 ± 0.62 ^a^	0.81 ± 0.69 ^a^	230.1 ± 41.10 ^a^	16.9 ± 1.52 ^a^	0.13 ± 0.04 ^a^
(12 h)	(5.23–6.45)	(11.1–14.5)	(30.0–44.9)	(6.60–24.4)	(3.0–17.4)	(0.10–2.40)	(0.10–3.20)	(198–356)	(14–21)	(0.05–0.22)
T6	5.96 ± 0.55 ^a^	12.4 ± 0.94	39.1 ± 3.77 ^a^	15.6 ± 2.65 ^a^	7.51 ± 3.80 ^a^	0.45 ± 0.48	0.40 ± 0.27	247.9 ± 49.34 ^a^	17.2 ± 1.62 ^a^	0.14 ± 0.05 ^a^
(24 h)	(5.05–6.94)	(10.7–14.3)	(31.4–45.9)	(10.6–20.9)	(3.08–18.1)	(0.10–1.80)	(0.0–1.00)	(195–357)	(15–22)	(0.03–0.21)
T7	6.28 ± 0.58	12.7 ± 0.94	40.1 ± 4.06	13.5 ± 3.52	5.28 ± 2.32	0.50 ± 0.31	0.38 ± 0.22	257.9 ± 55.23 ^a^	16.8 ± 1.07	0.16 ± 0.04 ^a^
(3 days)	(5.45–7.57)	(10.9–15.4)	(32.2–48.1)	(8.40–22.0)	(2.47–11.6)	(0.10–1.10)	(0.0–0.80)	(198–398)	(16–20)	(0.06–0.25)
T8	6.61 ± 0.61	12.8 ± 1.28	46.7 ± 3.87	9.97 ± 2.37	4.24 ± 1.43	0.42 ± 0.22	0.30 ± 0.22	317.9 ± 82.23 ^a^	16.6 ± 1.16	0.20 ± 0.05 ^a^
(7 days)	(5.67–7.92)	(11.2–16.4)	(36.4–52.2)	(5.70–14.4)	(2.43–8.29)	(0.0–0.90)	(0.0–0.80)	(201–472)	(14–19)	(0.08–0.28)

RBC (red blood cell); HB (hemoglobin concentration); PCV (packed cell volume); WBC (white blood cell); NFS (neutrophil); EOS (eosinophil); MON (monocyte); PLT (platelet); PDW (platelet distribution width) and PCT (plateletcrit).

**Table 2:** Pre-, intra- and post- mean ± SD of plasma glucose, BUN, triglycerides and phosphorus concentrations, and plasma CK activity in healthy bitches. Letter indicates significant differences vs. baseline values: ^a^ *p* < 0.05.

	GLU (mg/dL)	BUN (mg/dL)	TG (mg/dL)	P (mg/dL)	CK (UI/L)
T0	100.9 ± 19.4	30.3 ± 9.50	53.8 ± 22.2	4.29 ± 0.78	98.7 ± 37.4
(baseline)	(80–156)	(13.2–46.5)	(25–98)	(2.5–5.4)	(53–216)
T1	121.7 ± 28.8	26.7 ± 7.95 ^a^	33.8 ± 8.0	4.37 ± 0.82	97.1 ± 36.2
(30 min)	(81–180)	(15.9–46.2)	(24–56)	(2.9–5.4)	(53–187)
T2	173.5 ± 36.0 ^a^	25.7 ± 6.81 ^a^	32.26 ± 9.76 ^a^	4.72 ± 0.49 ^a^	111.7 ± 69.2
(60 min)	(111–231)	(12.7–45.8)	(26–56)	(3.7–5.4)	(53–381)
T3	154.2 ± 31.3 ^a^	24.9 ± 6.64 ^a^	35.9 ± 13.1 ^a^	4.20 ± 0.82 ^a^	130.4 ± 76.1
(3 h)	(90–212)	(14.5–47.0)	(26–77)	(2.6–5.6)	(68–342)
T4	121.1 ± 31.7 ^a^	24.5 ± 6.57 ^a^	34.1 ± 14.2 ^a^	4.18 ± 0.78	183.4 ± 88.8
(6 h)	(87–201)	(15.3–38.3)	(25–93)	(3.0–5.5)	(85–450)
T5	107.8 ± 21.2	22.8 ± 5.67 ^a^	33.4 ± 11.7	4.59 ± 0.84	279.7 ± 112.7 ^a^
(12 h)	(82–157)	(11.1–33.2)	(24–76)	(2.9–5.5)	(102–483)
T6	102.2 ± 15.7	19.1 ± 7.48 ^a^	45.1 ± 21.8	4.01 ± 0.82	452.7 ± 178.8 ^a^
(24 h)	(80–131)	(8.4–32.0)	(24–117)	(2.7–5.6)	(107–678)
T7	101.5 ± 10.6	19.1 ± 7.48 ^a^	43.3 ± 20.3	3.88 ± 0.74	473.2 ± 174.7 ^a^
(3 days)	(80–120)	(8.4–32.0)	(25–100)	(3.0–5.4)	(162–765)
T8	97.2 ± 11.4	27.4 ± 9.99	43.1 ± 16.5	4.26 ± 0.72	169.4 ± 99.6
(7 days)	(83–132)	(14.9–52.0)	(21–83)	(3.1–5.5)	(95–573)

GLU (glucose); BUN (blood urea nitrogen); TG (triglycerides); P (phosphorus) and CK (creatine kinase.

## Data Availability

The data presented in this study are available on request from the corresponding author.
